# Phylogeny and reclassification of *Aconitum* subgenus *Lycoctonum* (Ranunculaceae)

**DOI:** 10.1371/journal.pone.0171038

**Published:** 2017-01-31

**Authors:** Yu Hong, Yan Luo, Qi Gao, Chen Ren, Qiong Yuan, Qin-Er Yang

**Affiliations:** 1 Key Laboratory of Plant Resources Conservation and Sustainable Utilization, South China Botanical Garden, Chinese Academy of Sciences, Guangzhou, People’s Republic of China; 2 University of Chinese Academy of Sciences, Beijing, People’s Republic of China; 3 Xishuangbanna Tropical Botanical Garden, Chinese Academy of Sciences, Mengla, People’s Republic of China; 4 Guangxi Institute of Botany, Guangxi Zhuangzu Autonomous Region and Chinese Academy of Sciences, Guilin, People’s Republic of China; The National Orchid Conservation Center of China; The Orchid Conservation & Research Center of Shenzhen, CHINA

## Abstract

Phylogenetic analyses were performed using multiple nuclear (ITS and ETS) and chloroplast regions (*ndh*F-*trn*L, *psb*A-*trn*H, *psb*D-*trn*T, and *trn*T-*trn*L) to test the monophyly of *Aconitum* subgen. *Lycoctonum* (Ranunculaceae) and reconstruct the phylogenetic relationships within the subgenus. The subgenus as currently circumscribed is revealed to be polyphyletic. To achieve its monophyly, sect. *Galeata* and sect. *Fletcherum*, both being unispecific and each having a unique array of characters (the latter even having the aberrant base chromosome number of *x* = 6), must be removed from the subgenus. The subgenus *Lycoctonum* should thus be redefined to include only two sections, the unispecific sect. *Alatospermum* and the relatively species-rich sect. *Lycoctonum*. The section *Alatospermum*, which is both morphologically and karyologically in the primitive condition, is resolved as the first diverging lineage of the subgenus *Lycoctonum* clade. The monophyly of sect. *Lycoctonum* is strongly supported, but all the ten series currently recognized within the section are revealed to be para- or poly-phyletic. Five major clades are recovered within the section. We propose to treat them as five series: ser. *Crassiflora*, ser. *Scaposa*, ser. *Volubilia*, ser. *Longicassidata*, and ser. *Lycoctonia*. Thus, a formal reclassification of subgen. *Lycoctonum* is presented, which involves segregating both sect. *Galeata* and sect. *Fletcherum* from the subgenus as two independent subgenera within the genus *Aconitum*, reinstating one series (ser. *Crassiflora*) and abolishing six series (ser. *Laevia*, ser. *Longibracteolata*, ser. *Micrantha*, ser. *Ranunculoidea*, ser. *Reclinata*, and ser. *Umbrosa*) within sect. *Lycoctonum*. The series affiliation of some species within the section is adjusted accordingly.

## Introduction

*Aconitum* L. subgen. *Lycoctonum* (DC.) Peterm. (Ranunculaceae) consists of ca. 40 species distributed in Eurasia, northern Africa, and eastern North America [1]. It is different from its closest ally, subgen. *Aconitum*, by having perennial rhizomes (vs. tuberous roots) [1–3]. Another subgenus long recognized, subgen. *Gymnaconitum* (Stapf) Rapaics, which has annual taproots, was recently segregated as an independent genus of its own, i.e. *Gymnaconitum* (Stapf) Wei Wang & Z. D. Chen [4]. Many of the species in subgen. *Lycoctonum* are of potential medicinal value [5].

The first comprehensive classification of *Aconitum* subgen. *Lycoctonum* was proposed by Lauener and Tamura [2] and Tamura and Lauener [6] based on morphology, in which four sections and 11 series were recognized (Table 1). Later, Tamura [1] basically adopted this classification with only some minor changes in the arrangement order of the sections and series, and in the number of series as well. As shown in Table 1, he divided the subgenus into four sections: sect. *Alatospermum* Tamura, sect. *Galeata* Rapaics, sect. *Fletcherum* Tamura, and sect. *Lycoctonum* DC. The former three are all unispecific, including each the eastern Himalayan *A*. *novoluridum* Munz, the Kashmir Himalayan *A*. *moschatum* (Brühl ex Duthie) Stapf, and the eastern Himalayan *A*. *fletcheranum* G. Taylor, respectively. The section *Lycoctonum*, occurring nearly in the same area as subgen. *Lycoctonum*, comprises the remaining species which were subdivided into nine series: ser. *Micrantha* Steinb. ex Tamura & Lauener, ser. *Scaposa* W. T. Wang, ser. *Laevia* Tamura & Lauener, ser. *Reclinata* Tamura & Lauener, ser. *Volubilia* (Nakai) Tamura & Lauener, ser. *Longibracteolata* Steinb. ex Tamura & Lauener, ser. *Ranunculoidea* Steinb. ex Tamura & Lauener, ser. *Lycoctonia* Tamura & Lauener, and ser. *Longicassidata* (Steinb. ex Nakai) Tamura & Lauener. It is to be noted that Kadota [7] proposed ser. *Umbrosa* (Steinb. ex Nakai) Kadota to accommodate *A*. *mashikense* Kadota & Umezawa, *A*. *gigas* H. Lév. & Vaniot and *A*. *umbrosum* (Korsh.) Kom, the latter two of which had been previously placed in ser. *Lycoctonia* by Tamura and Lauener [6] and Tamura [1]. Later, Kadota [8] ascribed another six species from northern Japan to ser. *Umbrosa*, five of which were described as new by him.

**Table 1:** Historical classifications of *Aconitum* subgen. *Lycoctonum*.

Tamura and Lauener (1979)	Tamura (1995)
**subgen. *Lycoctonum***	**subgen. *Lycoctonum***
**sect. *Galeata*** (*A*. *moschatum*)	**sect. *Alatospermum*** (*A*. *novoluridum*)
**sect. *Fletcherum*** (*A*. *fletcheranum*)	**sect. *Galeata*** (*A*. *moschatum*)
**sect. *Alatospermum*** (*A*. *novoluridum*)	**sect. *Fletcherum*** (*A*. *fletcheranum*)
**sect. *Lycoctonum***	**sect. *Lycoctonum***
**ser. *Scaposa*** (*A*. *scaposum*, var. *chloranthum*, var. *patentipilum*, *A*. *cavaleriei*, *A*. *aggregatifolium*)	**ser. *Micrantha*** (*A*. *apetalum*, *A*. *sajanense*, *A*. *brevicalcaratum*, var. *lauenerianum*, *A*. *chrysotrichum*)
**ser. *Crassiflora*** (*A*. *crassiflorum*)	**ser. *Scaposa*** (*A*. *scaposum*, var. *chloranthum*, var. *patentipilum*, *A*. *cavaleriei*, *A*. *aggregatifolium*, *A*. *crassiflorum*, *A*. rilongense*)
**ser. *Laevia*** (*A*. *laeve*, var. *curvipilosum*)	**ser. *Laevia*** (*A*. *laeve*, var. *curvipilosum*)
**ser. *Reclinata*** (*A*. *reclinatum*)	**ser. *Reclinata*** (*A*. *reclinatum*)
**ser. *Volubilia*** (*A*. *alboviolaceum*, *A*. *loczyanum*, *A*. *pseudolaeve*, *A*. *quelpaertense*, *A*. *chrysopilum*, *A*. *pterocaule*, var. *albidum*, var. *glabrescens*, *A*. *pteropus*, *A*. *desoulavyi*)	**ser. *Volubilia*** (*A*. *alboviolaceum*, *A*. *loczyanum*, *A*. *pseudolaeve*, *A*. *quelpaertense*, *A*. *chrysopilum*, *A*. *pterocaule*, var. *albidum*, var. *glabrescens*, *A*. *pteropus*, *A*. *desoulavyi*, *A*. *finetianum*^#^, *A*. *longecassidatum*^#^)
**ser. *Longibracteolata*** (*A*. *sukaczevii*)	**ser. *Longibracteolata*** (*A*. *sukaczevii*)
**ser. *Micrantha*** (*A*. *apetalum*, *A*. *sajanense*)	**ser. *Ranunculoidea*** (*A*. *ranunculoides*, *A*. *ajanense*, *A*. *crassifolium*)
**ser. *Brevicalcarata*** (*A*. *brevicalcaratum*, var. *lauenerianum*)	**ser. *Lycoctonia*** (*A*. *septentrionale*, *A*. *moldavicum*, *A*. *wardii*, var. *hopeiense*. *A*. *angustius*, *A*. *orientale*, *A*. *iranshahrii*, *A*. *ranunculifolium*, *A*. *monticola*, *A*. *krylovii*, *A*. *puchonroenicum*, *A*. *umbrosum*, *A*. *gigas*, *A*. *vulparia*, *A*. *pauciflorum*, *A*. *leucostomum*^#^, *A*. wangyedianense*, *A*. *sinomontanum*^#^, *A*. shennongjiaense*)
**ser. *Longicassidata*** (*A*. *barbatum*, var. *puberulum*, *A*. *kirinense*, var. *australe*, *A*. *lasiostomum*)	**ser. *Longicassidata*** (*A*. *barbatum*, var. *puberulum*, *A*. *kirinense*, var. *australe*, *A*. *lasiostomum*)
**ser. *Ranunculoidea*** (*A*. *ranunculoides*, *A*. *ajanense*, *A*. *crassifolium*)	
**ser. *Lycoctonia*** (*A*. *septentrionale*, *A*. *moldavicum*, var. *sinomontanum*, *A*. *wardii*, var. *hopeiense*, *A*. *angustius*, *A*. *orientale*, *A*. *iranshahrii*, *A*. *ranunculifolium*, *A*. *monticola*, *A*. *krylovii*, *A*. *puchonroenicum*, *A*. *umbrosum*, *A*. *gigas*, var. *hondoense*, *A*. *vulparia*, *A*. *pauciflorum*)	

* Species referred to the series by their author(s), not by Tamura and Lauener (1979) and Tamura (1995).

^#^ Species reduced to synonyms by Tamura and Lauener (1979) but recognized as independent species by other authors: both *Aconitum finetianum* and *A*. *longecassidatum* as synonyms of *A*. *pterocaule*, *A*. *leucostomum* as a synonym of *A*. *wardii*, and *A*. *sinomontanum* as a synonym of *A*. *moldavicum*.

The circumscription of *Aconitum* subgen. *Lycoctonum* by Tamura [1] has been questioned by some authors. Kadota [9] transferred *A*. *fletcheranum* from this subgenus to his new subgenus, subgen. *Tangutica* (W. T. Wang) Kadota, and still maintained its independent sectional status, stating that the nectary blade of the petal in this species was not provided with a tubular portion. For the same reason he also considered that the inclusion of *A*. *moschatum* in subgen. *Lycoctonum* was highly doubtful, although he did not pinpoint further its systematic position [9]. Indeed, except for *A*. *fletcheranum* and *A*. *moschatum*, all the other species within subgen. *Lycoctonum* have a nectary blade provided with a tubular portion [1, 2, 6, 9]. The inclusion of *A*. *fletcheranum* within subgen. *Lycoctonum* seems also quite abnormal in terms of karyological characters. Most recently Hong et al. [10] reported the chromosome number of this species as 2*n* = 12. This count represents a new base chromosome number of *x* = 6 for the genus *Aconitum* which otherwise has a uniform base chromosome number of *x* = 8. The karyotype of *A*. *fletcheranum* is unique in *Aconitum*. There are two largest metacentric chromosome pairs in this species, whereas in all the other diploid taxa of the genus with available chromosomal data there is only one such chromosome pair, with the second largest pair being submetacentric [10].

Tamura’s [1] classification of the largest section (including ca. 40 species) within *Aconitum* subgen. *Lycoctonum*, i.e., sect. *Lycoctonum*, may also be problematic. For example, he placed *A*. *brevicalcaratum* (Finet & Gagnep.) Diels and *A*. *chrysotrichum* W. T. Wang in ser. *Micrantha* while *A*. *crassiflorum* Hand.-Mazz. in ser. *Scaposa*, but these three species, all occurring in the southern part (western Sichuan, northwestern Yunnan) of the Hengduan Mountains region in southwestern China, are morphologically most closely similar to each other [11, 12]. More importantly, they are all tetraploid (2*n* = 32) with almost identical karyotypes [10, 13]. Yuan and Yang [13] proposed that the three species, together with *A*. *rilongense* Kadota, also a tetraploid species from western Sichuan, should be placed in one and the same series.

Utelli et al. [14] used the chloroplast intergenic spacer *psb*A-*trn*H and nuclear ITS region to study the relationships of the *Aconitum lycoctonum* L. species complex from Europe and the Caucasus Mountains. No other molecular phylogenetic study focused on subgen. *Lycoctonum* has been made, although more or less species of the subgenus (all belonging to sect. *Lycoctonum*) were chosen as outgroups or placeholders in phylogenetic analyses of subgen. *Aconitum* [15, 16] or of the tribe Delphinieae [4, 17]. Significantly, the nine species sampled by Jabbour and Renner [17] and the 18 species sampled by Wang et al. [4] form a well-supported monophyletic clade in the phylograms obtained from combined *trn*L-F and ITS dataset by using maximum likelihood method. It is also noteworthy that the genus *Aconitum*, after the segregation of subgen. *Gymnaconitum* from it, is a monophyletic group, with the genera *Delphinium* L. and *Gymnaconitum* as its sister groups [4, 17]. This provides us a framework to probe further into the phylogeny of subgen. *Lycoctonum*.

In the present study, we sampled the majority of the known species within *Aconitum* subgen. *Lycoctonum* and used multiple nuclear (ITS and ETS) and chloroplast regions (*ndh*F-*trn*L, *psb*A-*trn*H, *psb*D-*trn*T, and *trn*T-*trn*L) to perform phylogenetic analyses on this subgenus. Our aims were to (1) test the monophyly of subgen. *Lycoctonum*, (2) reconstruct the phylogenetic relationships within the subgenus, and (3) provide a reclassification of the subgenus that is phylogeny driven.

## Materials and methods

### Taxon sampling

We sampled 61 accessions representing 41 species (ca. 87% of the recognized taxa, Table 1) which covered all the sections and series in *Aconitum* subgen. *Lycoctonum* (S1 Table). In order to settle the dispute over the phylogenetic relationships of *A*. *fletcheranum* and *A*. *moschatum*, we further sampled 34 species from subgen. *Aconitum*, which represent four of its five sections according to Tamura [1] (sect. *Austrokoreensia* Nakai, not included herein, is unispecific, including only *A*. *austrokoreense* Koidz. from southern Korea). Based on the results of Jabbour and Renner [17] and Wang et al. [4], *Gymnaconitum gymnandrum* (Maxim.) Wei Wang & Z.D. Chen, the single species in *Gymnaconitum*, and three species of *Delphinium* were selected as outgroups.

### DNA extraction, PCR-amplification, and sequencing

Total DNA was extracted from silica gel-dried leaf tissue or herbarium specimens, using the modified CTAB method [18] or using a DNeasy Plant Mini Kit (Qiagen). Six markers were employed in this study, including ITS, ETS, *psb*A-*trn*H, *psb*D-*trn*T, *trn*T-*trn*L and *ndh*F-*trn*L. All primers used for amplification and sequencing are given in Table 2. The whole internal transcribed spacer (ITS, including ITS1, 5.8S rDNA, ITS2) was amplified with universal primers ITS5 and ITS4 [19]. For some degraded DNA from poor quality herbarium tissue, ITS1 and ITS2 regions were amplified separately with additional primers (P2 and P4) as internal primers (Table 2). For chloroplast DNA regions, primers were designed using Primer Premier 5.0 based on the complete chloroplast genome of *Megaleranthis saniculifolia* Ohwi [20] (Table 2). Polymerase chain reaction (PCR) was performed in a total reaction (25 μL) containing 14.75 μL ddH_2_O, 5 μL 5 × PrimeSTAR^®^ Buffer (Mg^2+^ plus), 2 μL dNTP Mixture (2.5 mM), 0.5 μL of each primer (10 μM), 0.25 μL of PrimerSTAR^®^ HS DNA Polymerase (2.5 U/μL), and 2 μL of template DNA (10–40 ng). All reactions were carried out as follows: predenaturation at 95°C for 3 min followed by 35 cycles of denaturation at 94°C for 30 s, annealing at 53°C for 40 s, extension at 72°C for 50 s, and a final extension at 72°C for 8 min. PCR products were checked for length and concentration on 1.5% agarose gels and sent to Shanghai Invitrogen Biotechnology for commercial sequencing. Sequences were aligned using CLUSTALX v.2.1 [21, 22] with default settings and adjusted manually using Bioedit v.7.0.5 [23]. The complete concatenated alignment of these six regions has been deposited in TreeBASE (http://www.treebase.org; accession number: S20400), but regions of poly-A/T (≥10 replicates) and ambiguous alignment (nucleotide positions 87–104, 339–370, 428–443, 659–680, 685–694, 1331–1352, 1391–1400, 1404–1414, 1891–1902, 1956–1968) were excluded from the following analyses.

**Table 2:** A list of the primers used in this study.

Primer	Fragment	Sequence (5’-3’)	Reference
ITS4	ITS2	TCCTCCGCTTATTGATATGC	White et al. (1990)
P4	ITS2	ATTGCAGAATCCCGTGAACC	This study
ITS5	ITS1	GGAAGTAAAAGTCGTAACAAGG	White et al. (1990)
P2	ITS1	GCTACGTTCTTCATCGATGC	This study
18S-ETS	ETS	ACTTACACATGCATGGCTTAATCT	Baldwin et al. (1998)
ETS-R	ETS	TGATTTTGGGTTTTCGATCCACTAC	This study
Ndh1F	Partial *ndh*F-*trn*L	ATTGTTTCCGATTCACCAGCTCTTA	This study
Ndh1R	Partial *ndh*F-*trn*L	GCAACTCACTCAGTTTCACAACGAA	This study
Ndh2F	Partial *ndh*F-*trn*L	TCGTTGTGAAACTGAGTGAGTTGCT	This study
RTLjR	Partial *ndh*F-*trn*L	GAGCAGCGTGTCTACCGATT	This study
psbA	*psb*A-*trn*H	GTTATGCATGAACGTAATGCTC	Sang et al. (1997)
trnH2	*psb*A-*trn*H	CGCGCATGGTGGATTCACAATCC	Tate (2002)
DT-f1	Partial *psb*D-*trn*T	ACCTCATAGCATTTTCGGGAC	This study
DT-r2	Partial *psb*D-*trn*T	GATTTATCTGAAGGAAAAGGGGGAA	This study
TTL1F	Partial *trn*T-*trn*L	CTCTGAGCTAAGCGGGCTCGCATAA	This study
TTL1R	Partial *trn*T-*trn*L	CCCCCACCCTTTTGAATGAACACAG	This study

### Phylogenetic analyses

Phylogenetic analyses of the nrDNA datasets, the chloroplast datasets, and the combined datasets, were conducted using PAUP v.4.0b10 [24], GARLI (genetic algorithm for rapid likelihood inference) v.2.0 [25] and MrBayes v.3.2.1 [26]. Maximum parsimony (MP) searches were performed using heuristic search methods with tree bisection reconnection (TBR) branch swapping, and equal weighting of all characters. The analyses were repeated 1,000 times with a random order of sequence addition in an attempt to sample multiple islands of most parsimonious trees. Bootstrap tests were carried out to evaluate node support using 1,000 replicates with heuristic search settings identical to those for the original search. We determined the best-fit model of sequence evolution using the program Modeltest v.3.7 [27]. Maximum likelihood (ML) searches were carried out in GARLI v.2.0 using models selected by the Akaike information criterion (AIC) for each dataset. GARLI was run with eight replicates, using the default settings. The topology with the highest likelihood score was chosen as the best tree. For statistical support of branches, non-parametric bootstrap values were computed with 100 replicates, and support values were calculated using PAUP v.4.0b10. Bayesian inferences (BI) were conducted using the different models selected from Modeltest for each partition. Ten million generations were run to estimate parameters relating to sequence evolution and likelihood probabilities using a Markov chain Monte Carlo (MCMC) method. Trees were collected every 1000th generation. Convergence of runs was tested by inspecting whether the standard deviation of split frequencies of the runs was < 0.01 and by using the effective sample sizes (ESS) as calculated with Tracer v.1.4 [28], considering ESS values > 200 as good evidence. After removing 25% of the generations as burn in, a 50% majority rule consensus tree was calculated to generate a posterior probability for each node.

### Incongruence tests

To evaluate the congruence of datasets from different gene markers we employed the incongruence length difference (ILD) test [29] implemented in PAUP v.4.0b10 [24]. We used simple taxon addition, TBR branch swapping, and heuristic searches with 999 repartitions of the data. The ILD test was carried out with pairwise partition for each gene dataset as well as with the combined dataset. *P*-values below 0.05 were considered as evidence of significant incongruence [29].

As the ILD test suggested a significant difference between the cpDNA and nrDNA data, we visually compared the cpDNA and nrDNA trees and located five samples that were incongruently placed with strong support. These include *Aconitum apetalum* (Huth) B. Fedtsch. ex Steinb., two accessions of *A*. *barbatum* Pers. var. *barbatum* (ZY69 and GQ150), *A*. *fletcheranum*, and *A*. *gigas* var. *hondoense* Nakai ex Tamura & Lauener. Both Wilcoxon signed-ranks (WSR) test [30, 31] and approximately unbiased (AU) test [32] were further employed to assess the level of contribution of these samples to the conflict between the cpDNA and nrDNA data. *Aconitum moschatum* was of special interest in the phylogenetic position and thus also subjected to the WSR and AU tests, although its sister relationship to subgen. *Aconitum* in the nrDNA tree did not receive strong support. These six samples were first pruned from the original nrDNA and cpDNA datasets and then re-added individually. For each sample, the relationship inferred from one dataset was used as a constraint topology to test against the alternative one inferred from the other data-set. For WSR tests, PAUP v.4.0b10 [24] was employed to optimize the constraint topologies using MP approach. Before AU tests, GARLI v.2.0 [25] was used to optimize the constraint topologies, and then to calculate the site-log-likelihood values for both the best and the optimized constraint trees. The AU tests were implemented in CONSEL v.0.2 [33] using the default settings. *P*-values below 0.05 were considered to indicate significant differences.

## Results

### Phylogenetic analyses of chloroplast sequence data

There was no significant incongruence among the four chloroplast regions and the *p*-values resulting from the ILD test of pairwise sequences are shown in Table 3. Therefore, we combined all markers into a single dataset. For the combined cpDNA dataset, information of each aligned DNA data, tree statistics for the MP analysis and the best-fit model of each region are given in Table 4.

**Table 3:** *P*-values of the partition-homogeneity tests. The pruned datasets are reconstructed from the original datasets by excluding *Acontium apetalum*, two accessions of *A*. *barbatum* var. *barbatum* (ZY69 and GQ150), *A*. *fletcheranum*, *A*. *gigas* var. *hondoense*, and *A*. *moschatum*. Bold-faced values indicate rejection of the null hypothesis with 95% confidence.

Dataset of	*P*-value
ETS vs. ITS	0.088
*ndh*F*-trn*L vs. *psb*A*-trn*H	0.050
*ndh*F*-trn*L vs. *psb*D*-trn*T	0.174
*ndh*F*-trn*L vs. *trn*T*-trn*L	0.097
*psb*A*-trn*H vs. *psb*D*-trn*T	0.309
*psb*A*-trn*H vs. *trn*T*-trn*L	1.000
*psb*D*-trn*T vs. *trn*T*-trn*L	0.576
cpDNA vs. nrDNA (original datasets)	**0.001**
cpDNA vs. nrDNA (pruned datasets)	**0.001**

**Table 4:** Statistics of the nuclear and chloroplast sequence datasets.

	ITS	ETS	*ndh*F-*trn*L	*psb*A-*trn*H	*psb*D-*trn*T	*trn*T-*trn*L	nrDNA	cpDNA	Combined cpDNA and nrDNA
Number of taxa	97	83	99	98	81	68	98	99	99
Aligned length	670	318	944	301	638	417	988	2300	3288
No. variable characters	267	185	180	65	92	81	452	402	854
No. parsimony-informative characters	173	133	94	41	41	37	306	211	517
Tree length (steps)	523	394	265	93	110	91	929	555	1532
Consistency index (CI)	0.671	0.624	0.815	0.774	0.864	0.923	0.643	0.809	0.683
Retention index (RI)	0.921	0.871	0.922	0.909	0.943	0.965	0.900	0.919	0.895
Rescaled consistency index (RC)	0.618	0.544	0.751	0.704	0.814	0.891	0.578	0.744	0.611
Model	GTR+I+G	GTR+G	TIM+I+G	HKY+G	K81uf+G	K81uf			

**Fig 1:**
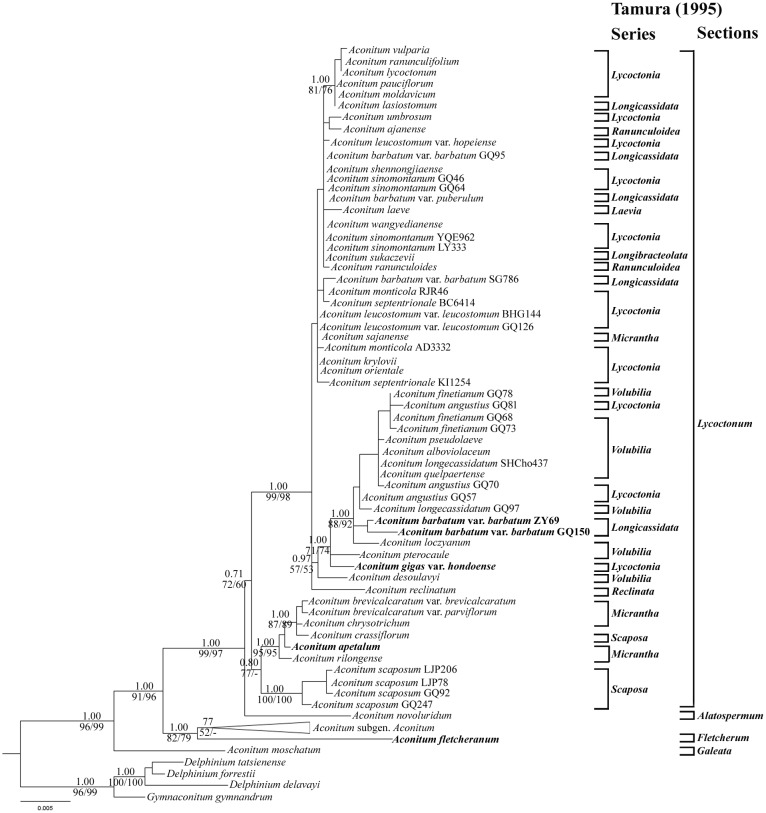
Phylogenetic relationships in *Aconitum* obtained from an ML analysis of the combined cpDNA dataset. Numbers above branches are posterior probabilities; numbers below branches are bootstrap values for maximum parsimony/maximum likelihood analyses. “-” indicates that support is less than 50% bootstrap value. Tamura’s (1995) classification of subgen. *Lycoctonum* are shown on the right. Accessions with a different placement in the nrDNA tree are indicated in bold. The clade of subgen. *Aconitum* has been collapsed for saving space (see S1 Fig for the complete topology).

The analyses of three approaches (MP, ML, and BI) revealed largely congruent tree topologies and the ML tree is shown in Fig 1 with support values. The monophyly of the genus *Aconitum* was strongly supported (PP/MP/ML = 1.00/96%/99%). However, the monophyly of subgen. *Lycoctonum* as currently circumscribed was not supported since its two members, *A*. *moschatum* (the single species of sect. *Galeata*) and *A*. *fletcheranum* (the single species of sect. *Fletcherum*), did not cluster together with other members in the same clade. *Aconitum moschatum* appeared to be sister to all the other species of *Aconitum* (PP/MP/ML = 1.00/91%/96%), whereas *A*. *fletcheranum* formed a clade together with species of subgen. *Aconitum* (PP/MP/ML = 1.00/82%/79%) and held a sister position to this subgenus, although with weak support (PP/MP = 0.77/52%). Except for *A*. *moschatum* and *A*. *fletcheranum*, all the remaining taxa of subgen. *Lycoctonum* formed a well-supported clade (PP/MP/ML = 1.00/99%/97%). *Aconitum novoluridum* (the single species of sect. *Altospermum*) was the first diverging lineage of the clade. The section *Lycoctonum* was resolved as a monophyletic group, although with somewhat weak support (PP/MP/ML = 0.71/72%/60%). It was further resolved as two clades. In one of them (PP/MP = 0.80/77%), two subclades with strong support were resolved: (1) four accessions of *A*. *scaposum* Franch. (PP/MP/ML = 1.00/100%/100%); (2) *A*. *brevicalcaratum* var. *brevicalcaratum*, *A*. *brevicalcaratum* var. *parviflorum* Chen & Liu, *A*. *chrysotrichum*, *A*. *crassiflorum*, *A*. *apetalum*, and *A*. *rilongense* (PP/MP/ML = 1.00/95%/95%). The phylogenetic relationships within the other clade (PP/MP/ML = 1.00/99%/98%) were largely unresolved, although *A*. *gigas* var. *hondoense*, *A*. *angustius* (W. T. Wang) W. T. Wang, and two accessions of *A*. *barbatum* var. *barbatum* formed a subclade together with species of ser. *Volubilia* with weak support (PP/MP/ML = 0.97/57%/53%), and six European species formed a subclade with strong support (PP/MP/ML = 1.00/81%/76%).

### Phylogenetic analyses of nuclear ribosomal DNA sequence data

The *p*-value resulting from the ILD test between nrDNA ETS dataset and nrDNA ITS dataset showed no significant incongruence (Table 3). We thus combined them in the phylogenetic analyses. For the combined nrDNA dataset, information of each aligned DNA data, tree statistics for the MP analysis and the best-fit model of each region are given in Table 4.

**Fig 2:**
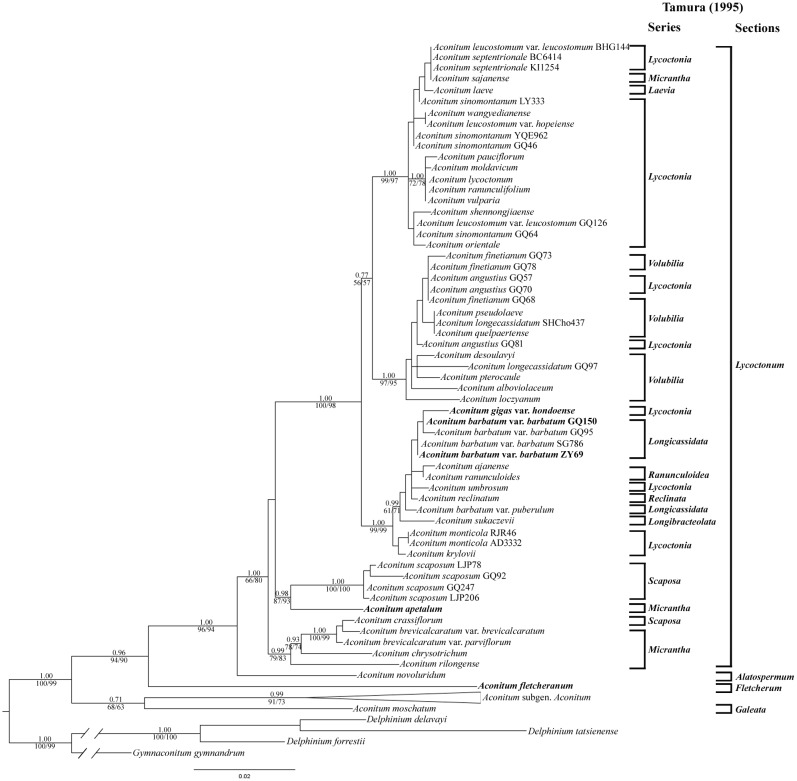
Phylogenetic relationships in *Aconitum* obtained from an ML analysis of the combined nrDNA dataset. Numbers above branches are posterior probabilities; numbers below branches are bootstrap values for maximum parsimony/maximum likelihood analyses. Tamura’s (1995) classification of subgen. *Lycoctonum* are shown on the right. Accessions with a different placement in the cpDNA tree are indicated in bold. The clade of subgen. *Aconitum* has been collapsed for saving space (see S2 Fig for the complete topology).

All the three analyses revealed approximately congruent tree topologies and the ML tree is shown in Fig 2 with support values. The monophyletic status of the genus *Aconitum* was confirmed with strong support values (PP/MP/ML = 1.00/100%/99%). However, as the case with cpDNA dataset, the monophyly of subgen. *Lycoctonum* as currently circumscribed was not supported. Its member *A*. *moschatum* was resolved as the sister to the subgenus *Aconitum* clade (PP/MP/ML = 0.99/91%/73%), although with relatively weak support (PP/MP/ML = 0.71/68%/63%). The remaining species of subgen. *Lycoctonum* formed a highly supported clade (PP/MP/ML = 0.96/94%/90%). Within this clade, *A*. *fletcheranum* and *A*. *novoluridum* were the first two species successively diverging. The section *Lycoctonum* was again resolved as a monophyletic group (PP/MP/ML = 1.00/66%/80%). Within this section, five clades with moderate to strong support were resolved. Comparison of the series classification by Tamura [1] on this section (Table 1) with our ML tree indicates that all the series recognized by him were para- or polyphyletic, although species of ser. *Volubilia* and of ser. *Lycoctonia* were almost nested together in a well-supported clade of their own respectively. The series *Umbrosa* was also not retrieved. It is noteworthy that the placements of *A*. *apetalum*, two accessions of *A*. *barbatum* var. *barbatum*, and *A*. *gigas* var. *hondoense* in the ML tree resulting from nrDNA dataset were different from their placements in the ML tree resulting from cpDNA dataset.

### Phylogenetic analyses of combined cpDNA and nrDNA data

The ILD test indicated strong incongruence between nuclear markers and chloroplast markers with a *p*-value of 0.001 (Table 3). The results of the WSR and AU tests are provided in Table 5. For *Aconitum gigas* var. *hondoense* and two accessions of *A*. *barbatum* var. *barbatum* (ZY69 and GQ150), all the tests suggested significant differences between the relationships inferred respectively from cpDNA and nrDNA datasets, indicating that they contributed greatly to the conflict. These samples were thus included in phylogenetic analyses as two entries, once as a cpDNA-only entry and once as an nrDNA-only entry. For *A*. *apetalum*, *A*. *fletcheranum* and *A*. *moschatum*, at least the tests of the nrDNA dataset showed no significant difference between the relationships inferred from the cpDNA and nrDNA datasets, suggesting that they should not be the main causes of the conflict between the two datasets. We thus combined their nrDNA and cpDNA sequences for phylogenetic analyses. However, it is to be noted that, even after excluding the six samples mentioned above, the *p*-value of the ILD test between the cpDNA and nrDNA datasets was still less than 0.001 (Table 4). A visual comparison showed that both the original (containing all the six samples) and pruned (containing only each of the six samples) datasets suggested the same positions for the six samples (results not shown here), indicating that their placements were not influenced by the other five samples.

**Table 5:** *P*-values of the WSR and AU tests. The pruned datasets are reconstructed from the original datasets by excluding *Aconitum apetalum*, two accessions of *A*. *barbatum* var. *barbatum* (ZY69 and GQ150), *A*. *fletcheranum*, *A*. *gigas* var. *hondoense*, and *A*. *moschatum*. Bold-faced values indicate rejection of the null hypothesis with 95% confidence.

Dataset	Constraint topology (inferred from the other dataset)	WSR	AU
Pruned nrDNA dataset			
+ *Aconitum moschatum*	*A*. *moschatum* is sister to all the remaining species of *Aconitum*	0.0956	0.311
+ *A*. *fletcheranum*	*A*. *fletcheranum* is sister to subgen. *Aconitum*	0.0578	0.559
+ *A*. *apetalum*	*A*. *apetalum* forms a clade with the tetraploid species from the Hengduan Mountains region	0.5637	0.307
+ *A*. *gigas* var. *hondoense*	*A*. *gigas* var. *hondoense* is a member of Clade C	**0.0010**	**0.011**
+ *A*. *barbatum* var. *barbatum* ZY69	*A*. *barbatum* var. *barbatum* ZY69 is a member of Clade C	**0.0033**	**<0.001**
+ *A*. *barbatum* var. *barbatum* GQ150	*A*. *barbatum* var. *barbatum* GQ150 is a member of Clade C	**0.0021**	**<0.001**
Pruned cpDNA dataset			
+ *A*. *moschatum*	*A*. *moschatum* is sister to subgen. *Aconitum*	**0.0253**	0.056
+ *A*. *fletcheranum*	*A*. *fletcheranum* is sister to subgen. *Lycoctonum*	0.1025	0.242
+ *A*. *apetalum*	*A*. *apetalum* is sister to *A*. *scaposum*	**0.0455**	**0.006**
+ *A*. *gigas* var. *hondoense*	*A*. *gigas* var. *hondoense* is a member of Clade D	**0.0384**	**<0.001**
+ *A*. *barbatum* var. *barbatum* ZY69	*A*. *barbatum* var. *barbatum* ZY69 is a member of Clade D	**0.0108**	**<0.001**
+ *A*. *barbatum* var. *barbatum* GQ150	*A*. *barbatum* var. *barbatum* GQ150 is a member of Clade D	**0.0108**	**<0.001**

**Fig 3:**
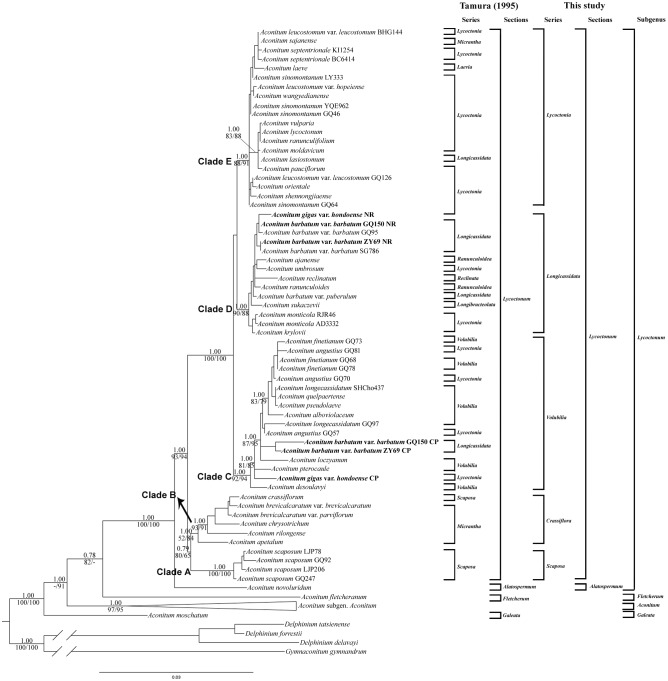
Phylogenetic relationships in *Aconitum* obtained from an ML analysis of the combined cpDNA and nrDNA dataset. Numbers above branches are posterior probabilities; numbers below branches are bootstrap values for maximum parsimony/maximum likelihood analyses. “-” indicates that support is less than 50% bootstrap value. Tamura’s (1995) classification and our new classification of subgen. *Lycoctonum* are shown on the right. Accessions with different placements between the cpDNA tree and the nrDNA tree are indicated in bold. The clade of subgen. *Aconitum* has been collapsed for saving space (see S3 Fig for the complete topology).

All the three analyses revealed approximately congruent tree topologies and the ML tree is shown in Fig 3 with support values. The genus *Aconitum* was confirmed again to be monophyletic with strong support values (PP/MP/ML = 1.00/100%/100%). The sister relationship of *A*. *moschatum* with the remaining species of *Aconitum* was supported by both BI and ML analyses (PP/ML = 1.00/91%). *Aconitum fletcheranum* was sister to subgen. *Lycoctonum* (PP/MP = 0.78/82%). The rest of species in subgen. *Lycoctonum* formed a well-supported clade (PP/MP/ML = 1.00/100%/100%). *Aconitum novoluridum* was the first split lineage. The monophyly of sect. *Lycoctonum* was well supported (PP/MP/ML = 1.00/93%/94%). Within this section, five major highly supported clades (Clades A–E) were resolved. These clades conformed largely to those revealed by the nrDNA dataset in taxon composition if regardless of those accessions with incongruent placements as revealed by nrDNA and cpDNA markers. All the series recognized by Tamura [1] were para- or poly-phyletic when superimposed on the ML tree (Fig 3), although Clade C consisted of taxa mostly from ser. *Volubilia* and Clade E was comprised of those mostly from ser. *Lycoctonia*. The series *Umbrosa* was also not recovered here, with its type species, *A*. *umbrosum*, deeply embedded in Clade D.

## Discussion

### Phylogenetic position and taxonomic status of *Aconitum* sect. *Fletcherum*

*Aconitum* sect. *Fletcherum* is unispecific, including only *A*. *fletcheranum* distributed in the eastern Himalayan region (Bhutan, southeastern Xizang in China, and Assam in northern India) [1–3, 9, 34–36]. When Tamura [34] established the section, he stated that the species was very peculiar in the short and scapose stem, the single flower terminal to the stem which was contrary to the indeterminate raceme in all other species of subgen. *Lycoctonum*, and in the navicular upper sepal and the 6–8 carpels as well. Lauener and Tamura [2], Wang [3], and Tamura [1] all accepted this section and its affiliation with subgen. *Lycoctonum*, but Kadota [9] transferred *A*. *fletcheranum* from subgen. *Lycoctonum* to his subgen. *Tangutica*, stressing that the nectary blade of the petal in this species, contrary to the other species (except for *A*. *moschatum*) within subgen. *Lycoctonum* but the same as members within subgen. *Tangutica*, was not provided with a tubular portion.

Most notably, the chromosome number of 2*n* = 12 in *Aconitum fletcheranum*, which represents a new base chromosome number of *x* = 6 for the genus *Aconitum* [10], is remarkably aberrant in the genus which otherwise has uniformly a base chromosome number of *x* = 8 [1, 13, 37–45]. Moreover, the karyotypic constitution of this species is also unique in the genus *Aconitum*. There are two largest metacentric chromosome pairs in this species, whereas in all the other diploid taxa of *Aconitum* there is only one such chromosome pair, with the second largest pair being submetacentric [10]. Hong et al. [10] considered that the base number of *x* = 6 in *Aconitum* may have originated from *x* = 8 (descending dysploidy) through asymmetric reciprocal translocations of some telocentric chromosomes. All these further suggest the abnormality of *A*. *fletcheranum* within subgen. *Lycoctonum*.

In our analyses, *Aconitum fletcheranum* is revealed to form a sister relationship to subgen. *Aconitum* in the cpDNA tree (PP/MP/ML = 1.00/82%/79% in Fig 1) and to all the remaining species of subgen. *Lycoctonum* in the nrDNA tree (PP/MP/ML = 0.96/94%/90% in Fig 2), respectively. There are several causes which may be invoked to account for this incongruence. All the topology tests show no significant difference between these two phylogenetic hypotheses, suggesting that stochastic errors, which are caused mainly by finite sequences used in studies, could not be ruled out confidently [46, 47]. Hybridization, probably combined with some other biological factors (reviewed in [48–52]), seems to be a more reasonable explanation for the incongruence of *A*. *fletcheranum* in view of its conflicting placements between the cpDNA and nrDNA trees, its aberrant chromosome number of 2*n* = 12 and unique karyotypic constitution. The signs of hybridization are nevertheless not evident in the nrDNA sequences of *A*. *fletcheranum*. We have failed to amplify the ETS sequence of *A*. *fletcheranum*, but we have successfully sequenced the ITS sequences of five individuals of this species. The chromatograms all contain well-formed, distinctive single peaks (no overlapping peaks) with very little background “noise”, and the sequences are different by only two nucleotides. It is regrettable that only one population of this species has been available for the present study. Sampling of more populations and further analyses using single- or low-copy nuclear genes are expected to reveal a more comprehensive and convincing pattern for this incongruence. Systematic errors, such as long-branch attraction, which result primarily from model misspecification [46], seem to be less likely a cause for the incongruence of *A*. *fletcheranum*. This is indicated by the results that, for each dataset, all the analyses (MP, ML, and BI) suggest the same placement for *A*. *fletcheranum* and that the branch length is generally even. Moreover, the positions of *A*. *fletcheranum* are stable in the cpDNA and nrDNA trees, at least not influenced by *A*. *apetalum*, the two accessions of *A*. *barbatum* var. *barbatum* (ZY69 and GQ150), *A*. *gigas* var. *hondoense*, and *A*. *moschatum*.

Our analyses of the combined data place *Aconitum fletcheranum* as a sister to all the remaining species of subgen. *Lycoctonum* (PP/MP = 0.78/82% in Fig 3), the same position as suggested by the nrDNA data. Although further studies are still needed to disentangle its evolutionary history, *A*. *fletcheranum* should deserve a subgeneric status in the genus *Aconitum* given that it always occupies a sister position to subgen. *Aconitum* (Fig 1) or to all the remaining taxa in subgen. *Lycoctonum* (Figs 2 and 3) and that it has a unique array of morphological and cytological characters.

Our molecular work lends no support to the establishment of *Aconitum* subgen. *Tangutica*. In all our analyses, *A*. *tanguticum* (Maxim.) Stapf, the type species of this subgenus, is always embedded in subgen. *Aconitum* (S1–S3 Figs), conforming to the results of previous molecular works on the genus *Aconitum* [15] or on the tribe Delphinieae [17]. Karyological data also indicate the membership of *A*. *tanguticum* within subgen. *Aconitum*. This species has the same chromosome number (2*n* = 16) and chromosome morphology as other taxa within subgen. *Aconitum* [44].

### Phylogenetic position and taxonomic status of *Aconitum* sect. *Galeata*

*Aconitum* sect. *Galeata* is also unispecific, including only *A*. *moschatum* endemic in the alpine zone of Kashmir [1, 2, 9, 34, 53]. Tamura [1, 34] and Lauener and Tamura [2] accepted this section and its affiliation with subgen. *Lycoctonum*, although they stated that *A*. *moschatum* was very particular within subgen. *Lycoctonum* by having the navicular or depressed galeate helmet broader than long, lurid purple flowers, and the very obtuse spur of the petal, not opposite to the labium but a continuation of the stalk. Kadota [9], however, pointed out that *A*. *moschatum* should not be placed in subgen. *Lycoctonum*, stressing that its nectary blade of the petal, contrary to the other species (except for *A*. *fletcheranum*) within the subgenus, was not equipped with a tubular portion. He left the problem aside for a further study. The chromosome number of *A*. *moschatum* was reported to be 2*n* = 16 [54] but regrettably the chromosome morphology has yet been unknown.

In our ML tree resulting from cpDNA dataset, *Aconitum moschatum* is revealed as the sister to all other members of the genus *Aconitum* with strong support (PP/MP/ML = 1.00/91%/96% in Fig 1). In the ML tree resulting from nrDNA dataset, however, it is resolved as the sister to subgen. *Aconitum*, but with weak support (PP/MP/ML = 0.71/68%/63% in Fig 2). All the topology tests, except for the WSR test of the cpDNA data, show no significant difference between the two phylogenetic hypotheses (Table 5). This may suggest that the incongruence of *A*. *moschatum* between cpDNA and nrDNA is not a “hard” one [55]. Similar to the case with *A*. *fletcheranum*, systematic errors cannot be invoked to account for this incongruence due to the stable positions suggested by all the analyses (the MP, ML, and BI analyses, and the analyses based on complete or pruned datasets) and the relatively short branch length as well. We thus combine the nrDNA and cpDNA data of *A*. *moschatum* for phylogenetic analyses. The analyses of combined data show that this species is the sister to all other species of *Aconitum* (PP/ML = 1.00/91% in Fig 3), the same relationship as suggested by cpDNA dataset.

In summary, our phylogenetic analyses indicate that *Aconitum moschatum* is not closely related to any of the major groups of *Aconitum* revealed here. Taking into account its unique array of morphological characters, we consider it justifiable to remove sect. *Galeata* from subgen. *Lycoctonum* and treat it as an independent subgenus of its own in the genus *Aconitum*.

### Phylogenetic position and taxonomic status of *Aconitum* sect. *Alatospermum*

*Aconitum* sect. *Alatospermum* is also unispecific, including only *A*. *novoluridum* distributed in the eastern Himalayan region (Bhutan, southeastern Xizang in China, Sikkim in northern India, and Nepal) [1–3, 34, 35]. When establishing the section under subgen. *Lycoctonum* based on this species, Tamura [34] pointed out that the species was clearly distinguishable from other species of the subgenus by the depressed hemi-elliptic helmet gradually descending into the long beak and much broader than long, the hammer-shaped petals with the nectary blade at a right angle to the stalk and longer than the stalk, the longitudinally winged seeds along the three ridges, and the lurid, reddish, brownish red or purple flowers. This section has since been recognized by Lauener and Tamura [2], Tamura [1], and Kadota [35] but rejected by Wang [3], who placed *A*. *novoluridum* in sect. *Lycoctonum* (“*Paraconitum*”).

Both the sectional status and subgeneric affiliation of *Aconitum novoluridum* are strongly supported by our molecular analyses. In our ML trees resulting from cpDNA (Fig 1), nrDNA (Fig 2) or the combined cpDNA and nrDNA dataset (Fig 3), this species is always resolved as the first split lineage in subgen. *Lycoctonum* and forms a sister relationship to sect. *Lycoctonum*.

Tamura [1] regarded *Aconitum* sect. *Alatospermum*, which has the seeds without transverse squamae and the hemi-elliptic upper sepal, as a primitive group in subgen. *Lycoctonum*, listing it as the first section in this subgenus (Tamura and Lauener [6] had previously listed sect. *Galeata* (“*Galeatum*”) as the first section). The primitive condition of *A*. *novoluridum* in seed morphology is confirmed by Kong et al. [56]. According to Hong et al. [10], the karyotype of *A*. *novoluridum* is the most symmetric in the subgenus and thus very probably represents a primitive condition. The primitive condition of *A*. *novoluridum* in subgen. *Lycoctonum* is also supported by our molecular work. This species is always the first split lineage in the subgenus in our ML trees resulting from different datasets.

Our molecular work, therefore, strongly favors the treatment of *Aconitum novoluridum* as an independent section within subgen. *Lycoctonum*, i.e. sect. *Alatospermum*, lending no support to its placement in sect. *Lycoctonum*. Evidence from morphology, karyology, and molecular phylogeny all suggests the primitive condition of sect. *Alatospermum* within subgen. *Lycoctonum*.

### Phylogenetic relationships within *Aconitum* sect. *Lycoctonum* and its reclassification

Our molecular work indicates that *Aconitum* sect. *Lycoctonum* is a monophyletic group within subgen. *Lycoctonum* (PP/MP/ML = 0.71/72%/60% in Fig 1; PP/MP/ML = 1.00/66%/80% in Fig 2; PP/MP/ML = 1.00/93%/94% in Fig 3), agreeing with the results of past molecular analyses involving this section [4, 17]. All the nine series recognized by Tamura [1] and ser. *Umbrosa* proposed additionally by Kadota [7] in this section, however, are revealed to be poly- or para-phyletic in our analyses of the combined cpDNA and nrDNA dataset (Fig 3). Five major clades are nevertheless recovered within the section, and they can be conveniently treated at series rank: ser. *Crassiflora*, ser. *Scaposa*, ser. *Volubilia*, ser. *Longicassidata*, and ser. *Lycoctonia*.

*Aconitum* ser. *Scaposa* includes only *A*. *scaposum* (Clade A: PP/MP/ML = 1.00/100%/100% in Fig 3). This species, fairly widespread in central and southwestern China [3] and recently reported, under the name *A*. *chloranthum* Hand.-Mazz., to occur also in Bhutan [35], shows great variation in the relative development of basal and cauline leaves, the leaf shape, pedicel pubescence, and flower color. Several varieties were once described in this species, and some of them were even recognized as independent species [2, 3, 57]. Yang [11] and Luo and Yang [12] treated *A*. *scaposum* as a polymorphic species including all the varieties and species. The four accessions that we have chosen in our molecular analyses represent two types of plants in respect of the relative development of basal and cauline leaves: two accessions (LJP206 from Sichuan, GQ92 from Gansu) have cauline leaves nearly aggregated in the middle part of the stem, while the other two accessions (GQ247 from Hubei, LJP78 from Chongqing) have cauline leaves nearly equally distantly arranged along the stem. All these accessions are nested together with each other in the same clade.

*Aconitum* ser. *Crassiflora* includes six taxa: *A*. *apetalum*, *A*. *brevicalcaratum* var. *brevicalcaratum*, *A*. *brevicalcaratum* var. *parviflorum*, *A*. *crassiflorum*, *A*. *chrysotrichum*, and *A*. *rilongense* (Clade B: PP/MP/ML = 1.00/52%/84% in Fig 3). The close affinity of the latter five taxa is also indicated by gross morphology, geographical distribution, and in particular, karyology. They are very similar to each other in general appearance [11, 12, 58] and all concentrated in the southern part of the Hengduan Mountains region in southwestern China [10]. They are all tetraploid (2*n* = 32) and share similar chromosome size and karyotype constitution [10]. The close affinity between *A*. *scaposum* (a diploid species with 2*n* = 16) and *A*. *crassiflorum* as previously regarded by Tamura [1] is not supported by our molecular analyses. In fact, no diploid species are clustered with these five tetraploid taxa. All the five tetraploid taxa in this series might have originated through only one polyploidization event, but we have been unable to ascertain their type of polyploidy (autopolyploidy vs. allopolyploidy) and parental origin from morphological, karyological and molecular data currently available.

*Aconitum apetalum* is distributed in central Asia (Xinjiang in China, Kazakhstan, and Tajikistan). It is readily distinguishable by its long, many-flowered raceme, very small flowers, and petals with a short, capitate spur, and the upper sepal narrowly cylindrical with a prominent peak [1, 6]. Tamura [1] placed it in ser. *Micrantha* together with *A*. *brevicalcaratum*, *A*. *chrysotrichum*, and *A*. *sajanense* Kumin mainly due to their short spur of petals, but in our molecular analyses *A*. *brevicalcaratum* and *A*. *chrysotrichum* are shown to be members in ser. *Crassiflora*, while *A*. *sajanense*, a diploid species with 2n = 16 [59], is shown to be a member in ser. *Lycoctonia* (Fig 3). The results of our molecular analyses of *A*. *apetalum* are somewhat similar to those of *A*. *fletcheranum*. As a hexaploid species (2*n* = 48), *A*. *apetalum* is nested with those tetraploid taxa (2*n* = 32) within ser. *Crassiflora* in the cpDNA tree (PP/MP/ML = 1.00/95%/95% in Fig 1) and sister to *A*. *scaposum* (2*n* = 16) in the nrDNA tree (PP/MP/ML = 0.98/87%/93% in Fig 2). This seems to strongly support a hybrid origin followed by subsequent polyploidization for *A*. *apetalum*. However, the chromatograms of ITS and ETS of *A*. *apetalum*, with only distinctive single peaks, show no obvious evidence of hybridization. The WSR and AU tests of the nrDNA data do not significantly reject the hypothesis inferred from the cpDNA data, further indicating the probable existence of a stochastic error. We thus also combine the cpDNA and nrDNA sequences of *A*. *apetalum* for phylogenetic analyses. The analyses place *A*. *apetalum* sister to all the tetraploid taxa from the Hengduan Mountains region, a relationship similar to that suggested by the cpDNA data. We therefore tentatively refer *A*. *apetalum* to ser. *Crassiflora*. It is worth mentioning that our molecular results do not support the reduction of *A*. *monticola* Steinb., also a central Asian species, to the synonymy of *A*. *apetalum* [60]. Both of them are nested in different clades (Fig 3).

*Aconitum* ser. *Volubilia* defined by Tamura and Lauener [6] and Tamura [1] is largely supported by our molecular work (Figs 1–3). Nine species traditionally classified in this series are clustered in this clade together with *A*. *angustius* (Clade C: PP/MP/ML = 1.00/92%/94% in Fig 3), a species mainly distributed in southeastern and central China and previously placed, under the name *A*. *sinomontanum* Nakai var. *angustius* W. T. Wang, in ser. *Lycoctonia* [6]. *Aconitum angustius* has long been considered to be most closely related to *A*. *sinomontanum* [57] or even treated as a variety of it [3, 61, 62]. This is not supported by our molecular results. The two taxa are nested in different clades (Fig 3). In fact, Gao et al. [63] and Hong et al. [10] has previously revealed that *A*. *angustius* is distinct from *A*. *sinomontanum* in ploidy level (tetraploid with 2*n* = 32 vs. diploid with 2*n* = 16) and that the former is morphologically more closely similar to *A*. *finetianum* Hand.-Mazz., a diploid species mainly distributed in southeastern China and placed (as a synonym of the Japanese species *A*. *pterocaule* Koidz.) in ser. *Volubilia* by Tamura and Lauener [6]. Moreover, as pointed out by Gao et al. [63], some specimens of *A*. *angustius* from Guizhou and Hubei, China, had been previously misidentified as *A*. *loczyanum* Rapaics or *A*. *pterocaule* (both belonging to ser. *Volubilia*) by Handel-Mazzetti [64] and Tamura and Lauener [6]. From its karyotypic constitution *A*. *angustius* may be of an allopolyploid origin [63]. Considering their close morphological similarity and somewhat overlapping geographical distribution we infer that one of the parents of *A*. *angustius* is very likely *A*. *finetianum*. The origin of *A*. *angustius* is an interesting problem worthy of further investigations.

*Aconitum* ser. *Longicassidata* (Clade D: PP/MP/ML = 1.00/90%/88% in Fig 3) is the most complex group which comprises species from five different series classified by Tamura [1]: ser. *Longibracteolata*, ser. *Longicassidata*, ser. *Lycoctonia*, ser. *Ranunculoidea*, and ser. *Reclinata*. The series *Umbrosa* proposed by Kadota [7] should also be transferred to here. Among them, ser. *Longibracteolata* and ser. *Reclinata* are both unispecific. The close similarity between *A*. *monticola* and *A*. *krylovii* Steinb. (ser. *Lycoctonia*) in morphology has been noted by Steinberg [65]. *Aconitum barbatum* and *A*. *kirinense* Nakai (ser. *Longicassidata*) have also been considered to be closely related to each other [6]; Handel-Mazzetti [64] even placed the latter in synonymy with the former. Most notably, while Tamura and Lauener [6] established ser. *Ranunculoidea* to accommodate *A*. *ranunculoides* Turcz. ex Ledeb. and *A*. *ajanense* Steinb., they pointed out that the latter species was also near to *A*. *umbrosum* (ser. *Lycoctonia*), a species which they considered to be closely related to *A*. *gigas* var. *hondoense* (= *A*. *iinumae* Kadota). These opinions are largely supported by our molecular results (Fig 3). *Aconitum reclinatum* A. Gray is the only representative of subgen. *Lycoctonum* in the New World [1, 6]. Our molecular phylogeny reveals it to be closely related to several Asian species, suggesting that it may have migrated from Asia to eastern North America.

It is noteworthy that two accessions of *Aconitum barbatum* var. *barbatum* (GQ150 and ZY69) and *A*. *gigas* var. *hondoense* show significant incongruences between the nrDNA and cpDNA datasets. The former taxon is widespread in northeastern and central China and the Far East of Russia (Primorye). Four accessions of it are included in our analyses, including one (GQ95) from Shaanxi in central China, two (GQ150 and ZY69) from Jilin in northeastern China, and one (SG786) from Primorye. On the nrDNA tree (Fig 2), the four accessions cluster together with *A*. *gigas* var. *hondoense* and then are nested within ser. *Longicassidata* (Clade D in Fig 3). On the cpDNA tree (Fig 1), GQ95 and SG786 are still grouped with species of ser. *Longicassidata* (although this series do not form a clade in the cpDNA tree), whereas GQ150 and ZY69 cluster with species of ser. *Volubilia* (Clade C in Fig 3). All the topology tests indicate significant incongruence between the two phylogenetic hypotheses of GQ150 and ZY69 (Table 5). However, both GQ150 and ZY69 are typical *A*. *barbatum* var. *barbatum* in morphology, showing no obvious difference from GQ95 and SG786, and no intermediates between *A*. *barbatum* var. *barbatum* and any species of ser. *Volubilia* have thus far been found. A visual examination of the nrDNA sequences of *A*. *barbatum* var. *barbatum* shows that there are no more than three nucleotide differences among its four accessions, and those of ZY69 (with incongruent placements) and SG786 (with congruent placement) are even totally identical. This seems to be a typical pattern of gene tree incongruence caused by chloroplast capture: the cytoplasm of GQ150 and ZY69 has been replaced by that of a certain member of ser. *Volubilia* probably via introgression [66–68]. This hypothesis is further indicated by the geographical distribution of *A*. *barbatum* var. *barbatum* and species of ser. *Volubilia*. Members in ser. *Volubilia* occur mainly in southeastern and northeastern China, the Korean Peninsula, Japan, and the Far East of Russia, with their distribution largely overlapping with that of *A*. *barbatum* var. *barbatum*. In the field *A*. *barbatum* var. *barbatum* is often found to grow in the neighborhood of some species of ser. *Volubilia*, e.g., *A*. *alboviolaceum* Kom. (pers. observ.).

Similar to the case with the two accessions of *Aconitum barbatum* var. *barbatum*, GQ150 and ZY69, *A*. *gigas* var. *hondoense* is nested in ser. *Longicassidata* on the nrDNA tree (Fig 2) but in ser. *Volubilia* on the cpDNA tree (Fig 1). The topology tests also indicate significant incongruence between the two phylogenetic hypotheses (Table 5). Regrettably only one accession of this variety is included in this study. A more extensive sampling is needed to explore the overall phylogenetic pattern of this taxon and the exact cause(s) of the incongruence of the accession sampled here. From a morphological perspective, *A*. *gigas* var. *hondoense* is closely related to *A*. *umbrosum*, and thus should be placed in ser. *Longicassidata* construed here.

*Aconitum* ser. *Lycoctonia* (Clade E: PP/MP/ML = 1.00/88%/91% in Fig 3) includes 14 taxa sampled here. Among them, 11 have been previously placed within this series by Tamura and Lauener [6] and Tamura [1], but the remaining three placed within other series by them, one each respectively in ser. *Micrantha* (*A*. *sajanense*), ser. *Laevia* (*A*. *laeve* Royle), and ser. *Longicassidata* (*A*. *lasiostomum* Reichb. ex Besser). Significantly, the eight European species sampled are all nested in this clade. Six of them (*A*. *lasiostomum*, *A*. *lycoctonum*, *A*. *moldavicum* Hacquet, *A*. *pauciflorum* Host, *A*. *ranunculifolium* Reichb., and *A*. *vulparia* Reichb. ex Spreng.) cluster together in a strongly supported subclade (PP/MP/ML = 1.00/83%/88% in Fig 3). Although Tamura and Lauener [6] placed *A*. *lasiostomum* in ser. *Longicassidata*, they noted that this species was not typical of the series and might approach ser. *Lycoctonia*. Utelli et al. [14], based on their analyses on the *A*. *lycoctonum* species complex from Europe and the Caucasus Mountains using the chloroplast intergenic spacer *psb*A-*trn*H and nuclear ITS region, also considered that *A*. *lasiostomum* should belong to ser. *Lycoctonia*.

## Taxonomic treatment

The first formal phylogeny-based classification of *Aconitum* subgen. *Lycoctonum* is presented below (also see Fig 3), which involves segregating both sect. *Galeata* and sect. *Fletcherum* from this subgenus as two independent subgenera of their own within the genus *Aconitum*, reinstating one series (ser. *Crassiflora*) and abolishing six (ser. *Laevia*, ser. *Longibracteolata*, ser. *Micrantha*, ser. *Ranunculoidea*, ser. *Reclinata*, and ser. *Umbrosa*) within sect. *Lycoctonum*. The series affiliation of some species within the section is adjusted accordingly. We include only the more significant synonyms, for a more complete synonymy see Tamura [1].

***Aconitum* subgen. *Galeata*** (Rapaics) Y. Hong & Q. E. Yang, **comb. & stat. nov.**

**Type.** *A*. *moschatum* (Brühl ex Duthie) Stapf

**Basionym.** *A*. sect. *Galeata* Rapaics in Növényt. Közlem. 6: 140. 1907.

**Description.** Stem usually unbranched with a few cauline leaves. Basal leaves 5–7-fid, segments obovate-cuneate, incised-dentate. Inflorescence racemiform, many- flowered. Flowers lurid-purple; upper sepal navicular or depressed galeate; spur of the petal very obtuse. Carpels 3. Seeds broadly squamate. chromosome number: *x* = 8.

**Species and Distribution.** Unispecific, endemic in the alpine zone of Kashmir.

**Note.** Our molecular work indicates that this subgenus is the earliest diverging lineage of *Aconitum* and sister to all the remaining species of the genus (Fig 3). Morphologically it is readily distinguishable from subgen. *Aconitum* by having rhizomes. From subgen. *Lycoctonum* it differs in the upper sepal navicular or depressed galeate, broader than long, and in the nectary blade of the petal not provided with a tubular portion. From subgen. *Fletcherum* it differs in the inflorescence racemiform, many-flowered.

***Aconitum* subgen. *Fletcherum*** (Tamura) Y. Hong & Q. E. Yang, **comb. & stat. nov.**

**Type.** *A*. *fletcheranum* G. Taylor

**Basionym.** *A*. sect. *Fletcherum* Tamura in Sci. Rep. Osaka Univ. 15: 30. 1966.

**Description.** Stem short, subscapose. Basal leaves rosulate, 5-partite, segments incised-lobate; cauline leaves usually 2, bract-like, 3-partite into entire lobes, remarkably sheathy at the base. Flower single, terminal to the stem; blue-violet; upper sepal navicular; spur of petal obtuse, labium inconspicuous. Carpels 6–8. Seeds unknown. Chromosome number: *x* = 6.

**Species and Distribution.** Unispecific, in the alpine zone of Bhutan, southeastern Xizang in China, and Assam in India.

***Aconitum* subgen. *Lycoctonum*** (DC.) Peterm. in Deutschl. Fl. 16. 1846.

**Type.** *A*. *lycoctonum* L.

**Basionym.** *A*. sect. *Lycoctonum* DC. in Syst. Nat. 1: 367. 1817.

**Description.** Stem branched or unbranched, leafy. Leaves palmatifid, palmatipartite, or palmatisect. Inflorescence racemose or paniculate. Flowers blue, purple, yellow or white; upper sepal hemielliptic, cylindrical, conical to tubular, usually longer than broad; spur of petal obtuse, capitate or elongate, long or short, or absent. Carpels 3. Seeds longitudinally alate along 3 ridges, hardly squamate or transversely squamate. Chromosome number: *x* = 8.

**Species and Distribution.** Approximately 44 species in two sections distributed in Eurasia, northern Africa, and eastern North America.

**Note.** The subgenus defined here includes only two sections, sect. *Alatospermum* and sect. *Lycoctonum*, not identical to the concept of previous authors. Tamura and Lauener [6] and Tamura [1], placed another two sections, sect. *Fletcherum* and sect. *Galeata*, in this subgenus.

Section 1. ***Aconitum* sect. *Alatospermum*** Tamura in Sci. Rep. Osaka Univ. 15: 30. 1966.

**Type.** *A*. *novoluridum* Munz

**Description.** Stem usually unbranched, leafy. Leaves 5-fid to -partite, segments incised-dentate. Inflorescence racemiform, many-flowered. Flowers lurid reddish or purple; upper sepal hemielliptic with a broad beak; petal hammer-shaped, spur opposite to the lamina, produced at right angles to the short, erect, thick stalk. Carpels 3. Seeds longitudinally alate along 3 ridges, hardly squamate.

**Species and Distribution.** Unispecific, in Bhutan, southeastern Xizang in China, Sikkim in India, and Nepal.

Section 2. ***Aconitum* sect. *Lycoctonum*** DC. in Syst. Nat. 1: 367.1817.

**Type.** *A*. *lycoctonum* L.

**Description.** Stem branched or unbranched, leafy. Leaves palmatifid, palmatipartite, or palmatisect. Inflorescence racemose or paniculate. Flowers blue, purple, yellow or white; upper sepal cylindrical, conical or high-galeate, usually longer than broad, often recurved at the apex; spur of petal obtuse, capitate or elongate, long or short, or absent. Carpels 3. Seeds transversely squamate.

**Species and Distribution.** Approximately 43 species in six series distributed in Eurasia, northern Africa, and eastern North America.

**Note.** At this stage it is very difficult to give an exact estimation of the number of species in this section because most of the species are most highly variable morphologically and different authors often have quite different concepts of species. Taxonomic revision at species level is still badly needed for some series of this section, e.g., ser. *Scaposa*, ser. *Volubilia*, ser. *Longicassidata*, and ser. *Lycoctonia*.

Series 1. ***Aconitum* ser. *Scaposa*** W. T. Wang in Acta Phytotax. Sin. Addit. 1: 60. 1965. **Type**. *A*. *scaposum* Franch.

**Description.** Stem scapose. Leaves reniform-pentagonal, 3-parted nearly to midvein, central lobe rhombic or cuneate-rhombic, lateral lobes obliquely flabellate, unequally 2-lobed. Inflorescence racemose, many-flowered, lax; pedicels long, usually spreading hairy; bracteoles 2, ovate or oblong, near the base of pedicels. Flowers purplish, greenish or pale-yellow; upper sepal cylindric; petaline lip linear, spur coiled, longer than lip.

**Species and Distribution.** Unispecific and yet highly polymorphic, in Bhutan and central and southwestern China.

Series 2. ***Aconitum* ser. *Crassiflora*** Tamura & Lauener in Notes Roy. Bot. Gard. Edinburgh 37: 123. 1979.

**Type**. *A*. *crassiflorum* Hand.-Mazz.

**Synonym**. *A*. ser. *Brevicalcarata* Tamura & Lauener in Notes Roy. Bot. Gard. Edinburgh 37: 443. 1979.

**Type**. *A*. *brevicalcaratum* (Finet & Gagnep.) Diels

**Synonym**. *A*. ser. *Micrantha* Steinb. ex Tamura & Lauener in Notes Roy. Bot. Gard. Edinburgh 37: 442. 1979.

**Type**. *A*. *apetalum* (Huth) B. Fedtsch. ex Steinb.

**Description.** Stem erect, scapose or subscapose, leafy, branched to inflorescences. Leaves obicular-reniform or reniform, 3-parted slightly beyond middle, central lobe cuneate-obtrapezoid, obovate-rhombic or rhombic, 3-fid, lateral lobes obliquely flabellate, unequally 2- or 3-fid. Inflorescence racemose, many-flowered; pedicels spreading or appressed pubescent; bracteoles 2, linear, usually near the base of pedicels. Flowers blue, blue-purple, dull yellow or yellowish; upper sepal high-galeate, cylindrical-galeate or cylindric; petal lip linear, conspicuous, spur slightly incurved, circinate or ecalcarate.

**Species and Distribution.** Five species, *A*. *apetalum*, *A*. *brevicalcaratum*, *A*. *chrysotrichum*, *A*. *crassiflorum*, and *A*. *rilongense*, in the southern part (western Sichuan and northeastern Yunnan) of the Hengduan Mountains region in southwestern China, Xinjiang, China and Kazakhstan.

Series 3. ***Aconitum* ser. *Volubilia*** (Steinb. ex Nakai) Tamura & Lauener in Notes Roy. Bot. Gard. Edinburgh 37: 434. 1979.

**Type.** *A*. *alboviolaceum* Kom.

**Description.** Stem erect, decumbent or twining. Leaves pentagonal-reniform, 3-parted slightly beyond middle, central lobe rhombic-obtrapezoid or broadly rhombic, lateral lobes obliquely flabellate, unequally 2- or 3-fid. Inflorescence racemose, simple or branched, elongate or more or less aggregate; pedicels short, spreading or appressed pubescent; bracteoles 2, linear, below the middle or near the base of pedicels. Flowers dilute purple or white; upper sepal cylindrical, more or less recurved at the tip; petal lip linear, conspicuous, spur curved or coiled, longer than the lip.

**Species and Distribution.** Approximately 11 species, mainly in eastern Asia (China, Korea, and Japan), e.g., *Aconitum alboviolaceum*, *A*. *angustius*, *A*. *desoulavyi* Kom., *A*. *finetianum*, *A*. *locyzanum* Rapcs., *A*. *longecassidatum* Nakai, *A*. *pseudolaeve* Nakai, *A*. *pterocaule*, and *A*. *quelpaertense* Nakai.

**Note.** This series has been fairly well defined previously by Tamura and Lauener [6] and Tamura [1]. Although *A*. *pteropus* Nakai, a species from Korea, is not included in our molecular analyses because of unavailability of DNA material, we agree with Tamura and Lauener [6] that it should belong to this series from a morphological perspective. Morphologically this species is closely similar to *A*. *pterocaule*.

Series 4. ***Aconitum* ser. *Longicassidata*** (Steinb. ex Nakai) Tamura & Lauener in Notes Roy. Bot. Gard. Edinburgh 37: 444. 1979.

**Type.** *A*. *barbatum* Pers.

**Synonym.** *A*. ser. *Reclinata* Tamura & Lauener in Notes Roy. Bot. Gard. Edinburgh. 37: 434. 1979. **syn. nov.**

**Type**. *A*. *reclinatum* A. Gray

**Synonym.** *A*. ser. *Logibracteolata* (Steinb. ex) Tamura & Lauener in Notes Roy. Bot. Gard. Edinburgh 37: 442. 1979. **syn. nov.**

**Type.** *A*. *sukaczevii* Steinb.

**Synonym.** *A*. ser. *Ranunculoidea* (Steinb. ex) Tamura & Lauener in Notes Roy. Bot. Gard. Edinburgh 37: 449. 1979. **syn. nov.**

**Type.** *A*. *ranunculoides* Turcz. ex Ledeb.

**Synonym.** *A*. ser. *Umbrosa* (Steinb. ex Nakai) Kadota in Fl. Jpn. IIa: 268. 2006. **syn. nov.**

**Type.** *A*. *umbrosum* (Korsh.) Kom.

**Description.** Stem erect, subscapose or leaning, sometimes trailing. Leaves obicular-reniform or reniform-pentagonal, 3-parted or subpedatifid to partite, central leaf segment broadly rhombic or cuneate-rhombic, sometimes 3-parted nearly to midvein, lateral segments obliquely flabellate. Inflorescence lax or densely racemiform; pedicels spreading or appressed pubescent; bracteoles below the middle or near the base of pedicels. Flowers yellow, white or pale blue-violet; upper sepal elongate conical, cylindrical or tubulose, often with a beak; petal lip linear, conspicuous, spur short or elongate, circinate or semi-coiled, shorter than, nearly as long as or longer than the lip.

**Species and Distribution.** Approximately 11 species, in eastern and central Asia and eastern North America, e.g., *Aconitum ajanense*, *A*. *barbatum*, *A*. *gigas*, *A*. *krylovii*, *A*. *monticola*, *A*. *ranunculoides*, *A*. *reclinatum*, *A*. *sukaczevii* Steinb., and *A*. *umbrosum*.

**Note.** This series is greatly expanded to include several species which have been previously placed in four other series by Tamura and Lauener [6] and Tamura [1]: *Aconitum reclinatum* (the single species in ser. *Reclinata*), *A*. *sukaczevii* (the single species in ser. *Longibracteolata*), *A*. *ajanense*, *A*. *ranunculoides* (both in ser. *Ranunculoidea*), *A*. *umbrosum*, *A*. *monticola* and *A*. *krylovii* (all in ser. *Lycoctonia*). Another two species, including *A*. *crassifolium* Steinb. (ser. *Ranunculoidea*) from the Far East of Russia and *A*. *puchonroenicum* Uyeki & Satake (ser. *Lycoctonia*) from Korea, should also belong to this series, but this needs to be verified by using molecular data.

Among the nine species (*Aconitum asahikawaense* Kadota, *A*. *gigas*, A. *hiroshi-igarashii* Kadota, *A*. *ikedae* Kadota, *A*. *mashikense*, *A*. *soyaense* Kadota, *A*. *tatewaki* Miyabe, *A*. *umezawae* Kadota, *A*. *umbrosum*) placed by Kadota [7, 8] in his ser. *Umbrosa*, only *A*. *gigas* var. *hondoense* and *A*. *umbrosum* are included in our molecular analyses. It is somewhat strange to us that when Kadota [8] described *A*. *hiroshi-igarashii* as new, he compared it with both *A*. *pterocaule* and *A*. *gigas*, the former of which is a member of ser. *Volubilia*. Judging from their morphological characters, *A*. *tatewaki* and all the above-mentioned species described as new by Kadota are very closely related to *A*. *gigas*, and their identities need further determination.

Series 5. ***Aconitum* ser. *Lycoctonia*** Tamura & Lauener in Not. Bot. Gard. Edinb. 37: 451. 1979.

**Type.** *A*. *lycoctonum* L.

**Synonym.** *A*. ser. *Laevia* Tamura & Lauener in Notes Roy. Bot. Gard. Edinburgh 37: 433. 1979. **syn. nov.**

**Type.** *A*. *laeve* Royle

**Description.** Stem erect, sometimes decumbent. Leaves obicular-reniform, 3-parted or subpedatifid to partite, central lobe broadly rhombic or narrowly cuneate-rhombic, 3-fid, lateral lobes obliquely flabellate, unequally 3-fid. Inflorescence racemose, many flowered; pedicels spreading or appressed pubescent; bracteoles 2, below the middle or near the base of pedicels. Flowers yellow, purple or blue; upper sepal elongate conical or cylindrical; petal lip linear, conspicuous, spur elongate, curved or circinate, nearly as long as or longer than the lip.

**Species and Distribution.** Approximately 15 species, in Europe, Asia and northern Africa, e.g., *Aconitum laeve*, *A*. *lasiostomum*, *A*. *leucostomum* Vorosh., *A*. *lycoctonum*, *A*. *moldavicum*, *A*. *orientale* Mill., *A*. *pauciflorum*, *A*. *ranunculifolium*, *A*. *sajanense*, *A*. *septentrionale*, *A*. *shennongjiaense* Q. Gao & Q.E. Yang, *A*. *sinomontanum*, *A*. *vulparia*, and *A*. *wangyedianense* Y.Z. Zhao.

**Note.** Although the Iranian species *Aconitum iranshahri* H. Riedl is not included in our molecular analyses, it should belong to this series from its close morphological similarity with *A*. *orientale*.

## Supporting information

S1 Fig Phylogenetic relationships in *Aconitum* obtained from an ML analysis of the combined cpDNA dataset. Numbers above branches are posterior probabilities; numbers below branches are bootstrap values for maximum parsimony/maximum likelihood analyses. “-” indicates that support is less than 50% bootstrap value.
(JPG)

S2 Fig Phylogenetic relationships in *Aconitum* obtained from an ML analysis of the combined nrDNA dataset. Numbers above branches are posterior probabilities; numbers below branches are bootstrap values for maximum parsimony/maximum likelihood analyses.
(JPG)

S3 Fig Phylogenetic relationships in *Aconitum* obtained from an ML analysis of the combined cpDNA and nrDNA dataset. Numbers above branches are posterior probabilities; numbers below branches are bootstrap values for maximum parsimony/maximum likelihood analyses. “-” indicates that support is less than 50% bootstrap value.
(JPG)

S1 Table Table of accessions, showing all individuals used in this study. (XLSX)
