# Supplementary figures and images for: Phylogeny and reclassification of Aconitum subgenus Lycoctonum (Ranunculaceae)
Source: PLoS One. 2017 Jan 31;12(1):e0171038. doi: 10.1371/journal.pone.0171038 (PMC5334035; doi:10.1371/journal.pone.0171038)

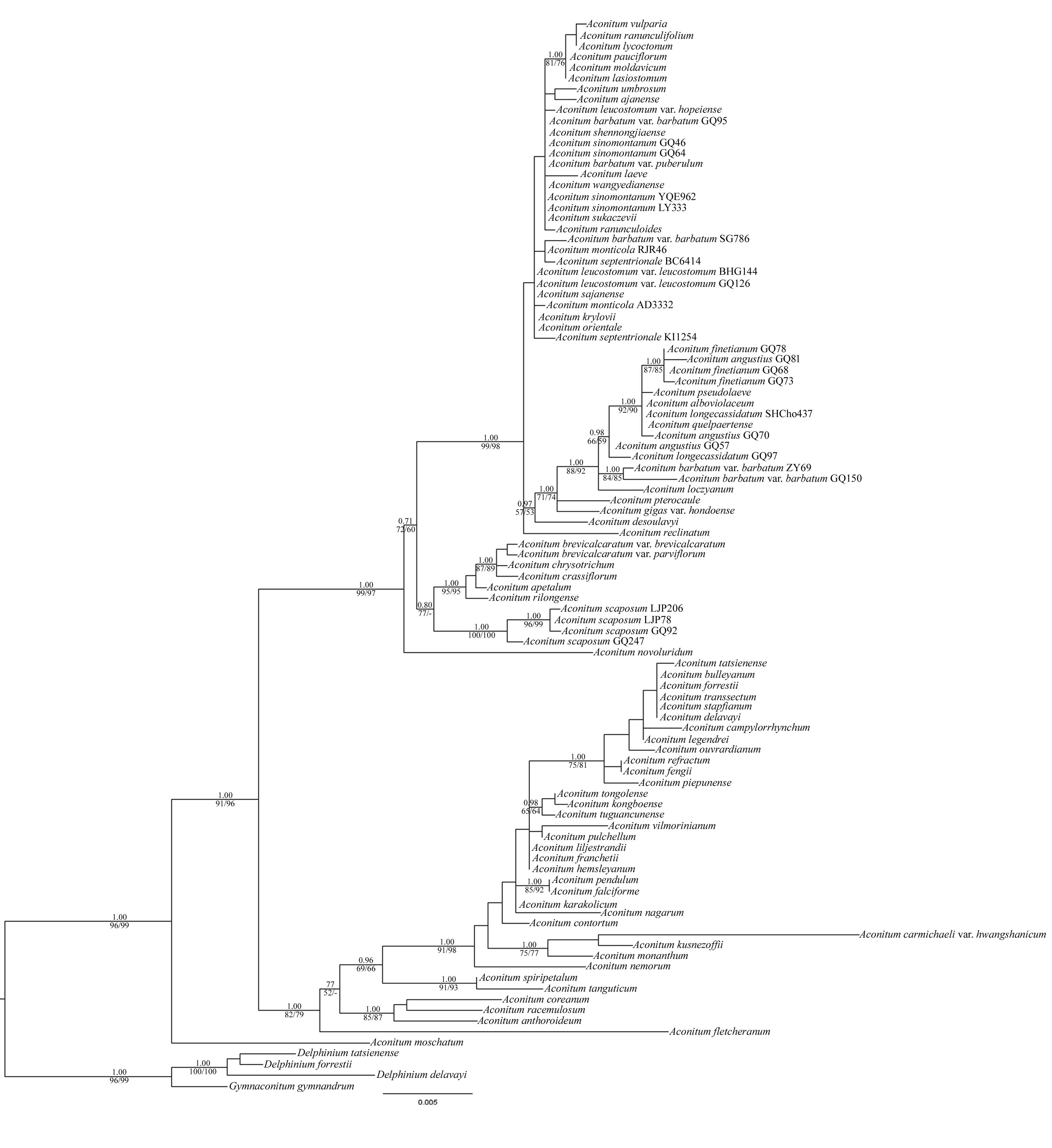

Supplement: S1 Fig — Numbers above branches are posterior probabilities; numbers below branches are bootstrap values for maximum parsimony/maximum likelihood analyses. “-” indicates that support is less than 50% bootstrap value. (JPG) [file pone.0171038.s001.jpg]

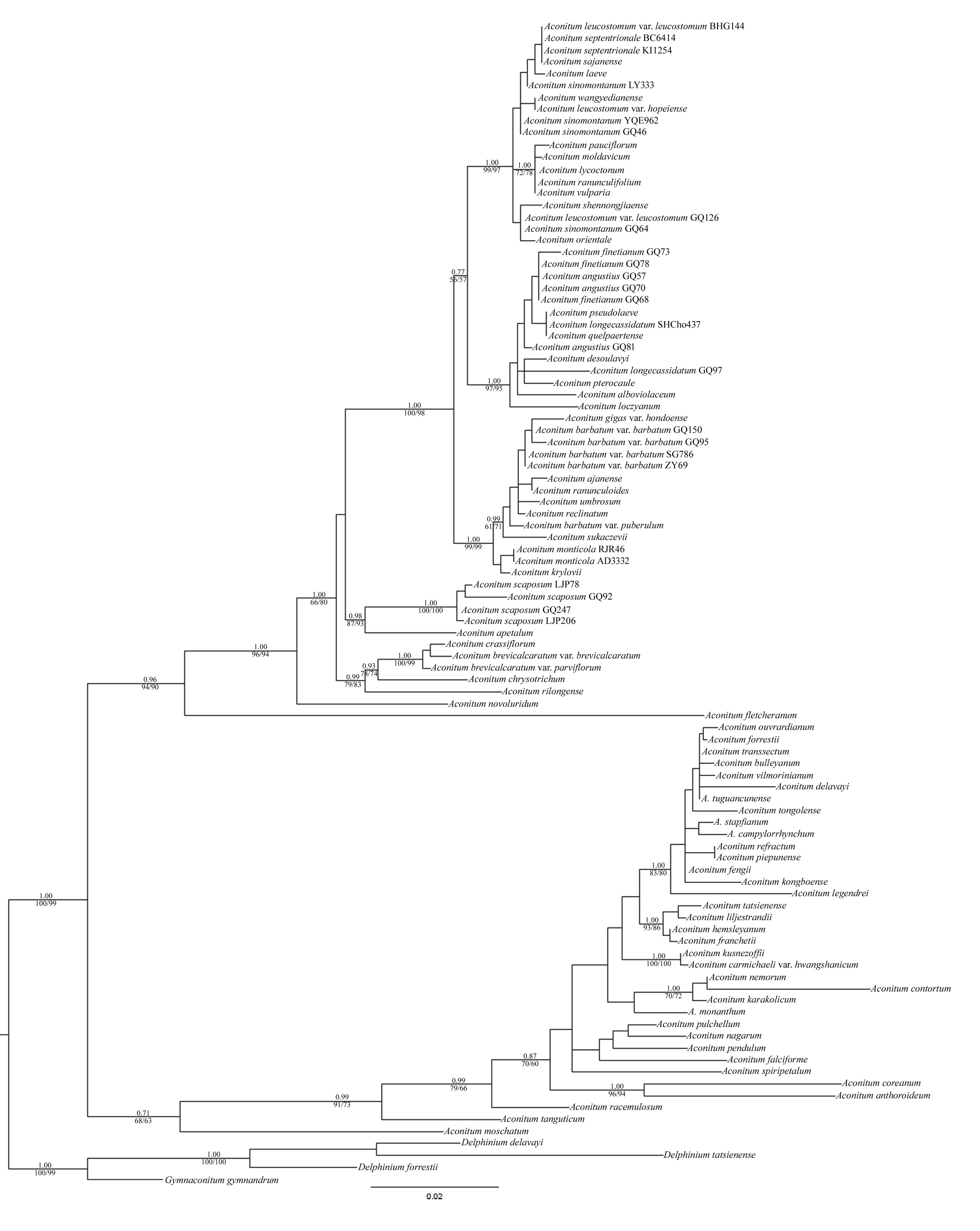

Supplement: S2 Fig — Numbers above branches are posterior probabilities; numbers below branches are bootstrap values for maximum parsimony/maximum likelihood analyses. (JPG) [file pone.0171038.s002.jpg]

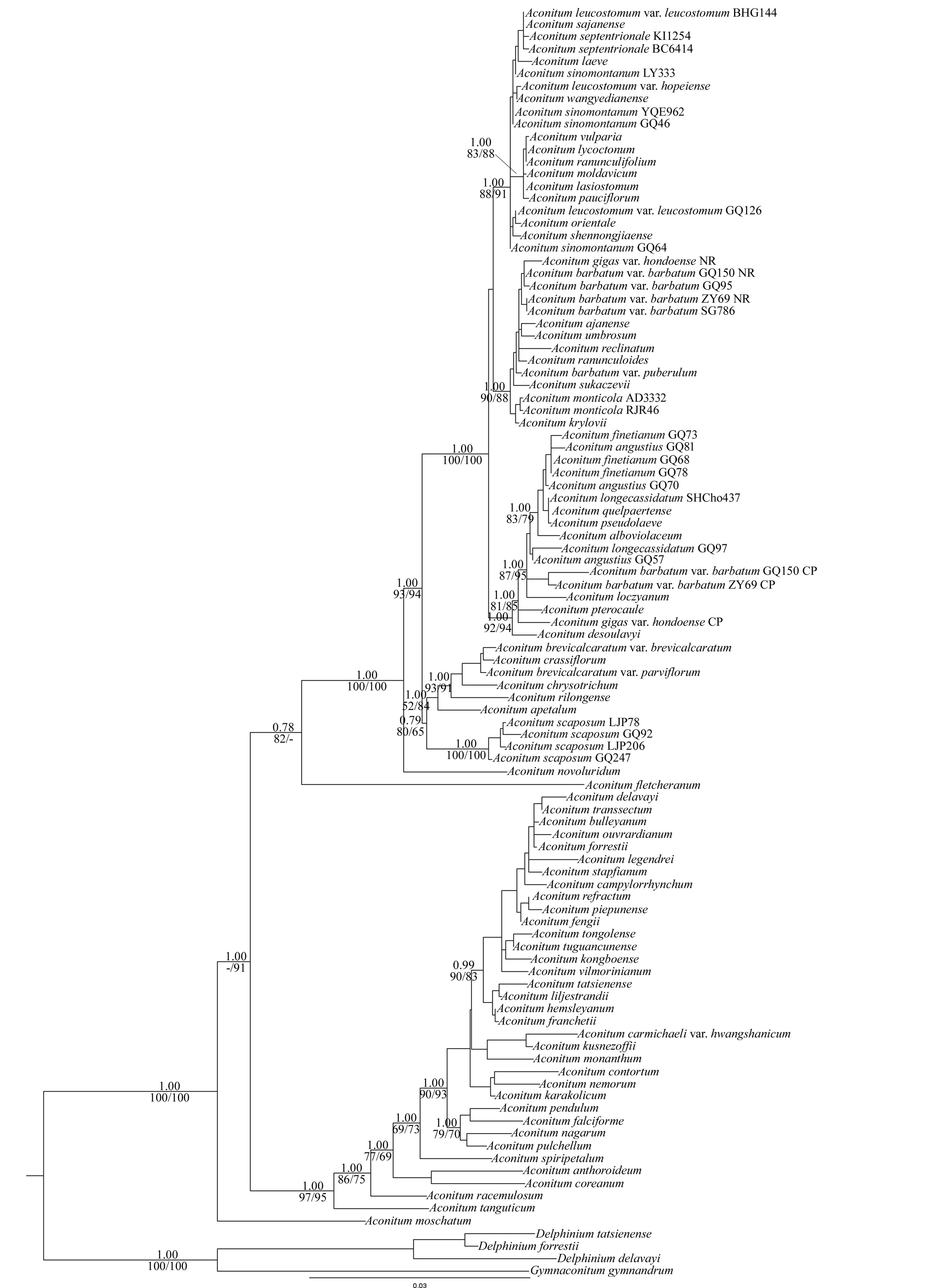

Supplement: S3 Fig — Numbers above branches are posterior probabilities; numbers below branches are bootstrap values for maximum parsimony/maximum likelihood analyses. “-” indicates that support is less than 50% bootstrap value. (JPG) [file pone.0171038.s003.jpg]
